# Matrix Interference of Vegetable on Enzyme-Linked Immunosorbent Assay for Parathion Residue Detection

**DOI:** 10.3390/foods14193414

**Published:** 2025-10-03

**Authors:** Linglong Chen, Ge Chen, Xing Zhang, Qinghuan Wu, Guangyang Liu, Xiaomin Xu, Yanguo Zhang, Lingyun Li, Lin Qin, Jing Wang, Maojun Jin, Donghui Xu

**Affiliations:** 1 Institute of Vegetables and Flowers, Chinese Academy of Agricultural Sciences, State Key Laboratory of Vegetable Biobreeding, Key Laboratory of Vegetable Quality and Safety Control, Ministry of Agriculture and Rural Affairs of China, Ministry of Agriculture Vegetable Product Quality Safety Risk Assessment Laboratory, Beijing 100081, China; 15581330584@163.com (L.C.); neve0522@gmail.com (X.Z.); wuqinghuann@163.com (Q.W.); liuguangyang@caas.cn (G.L.); xuxiaomin@caas.cn (X.X.); zhangyanguo@caas.cn (Y.Z.); lilingyun@caas.cn (L.L.); qinlin01@caas.cn (L.Q.); 2 Chongqing Key Laboratory of Olericulture, College of Horticulture and Landscape Architecture, Southwest University, Chongqing 400715, China; 3 Institute of Quality Standard and Testing Technology for Agro-Products, Chinese Academy of Agricultural Sciences, Key Laboratory of Agro-Product Quality and Safety, Ministry of Agriculture and Rural Affairs of China, Beijing 100081, China; w_jing2001@126.com (J.W.); katonking@163.com (M.J.)

**Keywords:** matrix interference, immunoassay, parathion residues, rapid detection, vegetable

## Abstract

Complex matrix of vegetable severely interferes with enzyme-linked immunosorbent assay (ELISA) accuracy, limiting its application in parathion residue detection. This study investigated the interference mechanism of vegetable matrix, including chlorophyll, perilla protein, glucose, fructose, and sucrose, on ELISA. Furthermore, we validated the vegetable matrix interference on parathion residue ELISA by comparing the matrix interference index (I_m_) and recovery rate of vegetable samples before and after acetic acid-treatment. The results demonstrate that the addition of vegetable matrix significantly interferes with ELISA, with the antibody–IgG-HRP binding being subject to the most pronounced interference. Compared to the I_m_ (16–26%) of non-acetic acid treatment, the I_m_ (10–13%) was significantly reduced after the acetic acid treatment. Concomitantly, spiked recovery experiments of acid-treated samples yielded satisfactory average recovery rate (80–113%) as the matrix interference was minimized. The findings of this study provide valuable insights into the mechanism of vegetable matrix interference on ELISA.

## 1. Introduction

The relentless growth of the global population exerts inevitable impacts on agricultural systems, with the vegetable industry facing particular pressure to increase yields. Increasingly high vegetable yields are required to meet escalating dietary demands. To guarantee vegetable yields, pesticides are typically sprayed during vegetable production to mitigate economic losses caused by pests and diseases [1]. Organophosphorus pesticides find widespread application in vegetable production due to their low phytotoxicity to plants and high efficacy. Globally, organophosphate pesticides account for 33% of total pesticide usage [2]. Parathion, a highly toxic organophosphate pesticide, is widely used for pest control in vegetable production [3,4]. Although both China and the EU have banned parathion and set strict maximum residue limits (0.01 mg/kg, GB 2763-2021 and EC No 396/2005) [5,6] for enhanced control, incidents of illegal use still occur [7,8]. Residues of parathion in vegetables pose severe health threats and environmental risks to humans [4,8]. Therefore, efficient, sensitive, and rapid detection of parathion residues is critical for food safety and environmental protection.

Currently, several instrument-based detection methods for parathion residues in vegetables have been developed, such as gas chromatography [9], liquid chromatography [10], gas chromatography–mass spectrometry (GC-MS) [11], and liquid chromatography–mass spectrometry (LC-MS) [12]. These techniques are characterized by low limits of detection and high sensitivity, but they require sophisticated instrumentation, specialized expertise, and lengthy procedures, limiting their applicability for on-site, large-scale screening. In contrast, enzyme-linked immunosorbent assay (ELISA) based on antigen−antibody specific recognition, exhibits the advantages of strong specificity, simple operation, and intuitive detection results. Consequently, ELISA has been widely used in pesticide residue detection [13].

Despite these advantages, the sensitivity and accuracy of ELISA are frequently compromised by various matrices in the samples [14]; the study of matrix interference on ELISA becomes an essential part of pesticide residue analysis. Most research on matrix interference predominantly focuses on instrumental detection methods [15,16,17,18]. With the wide application of ELISA in the field of pesticide residue detection, an increasing number of scholars have attempted to enhance ELISA performance through the incorporation of pre-treatment [19,20], optimization of signal components [21,22], and specific components [23,24,25]. While these approaches have improved ELISA capability of antimatrix interference, most are hampered by narrow operational stability and relatively high costs. Employing effective and straightforward methods to minimize matrix interference can promote the further application of ELISA in complex matrix samples.

Furthermore, while most current studies have focused on the improvement of ELISA, few studies have reported the interference mechanism of matrices on ELISA. Investigating the interference mechanism of matrices is indispensable to the reliable application of ELISA in diverse samples. Pesticide residue ELISA typically utilizes a monoclonal antibody for recognition and IgG-HRP for signal output. The accuracy of ELISA can be interfered by matrix at critical stages: antigen–antibody binding, antibody–IgG-HRP binding, and catalytic activity of HRP. While the interference mechanism of certain matrices on ELISA has been preliminarily explored [26,27,28], the interference mechanism of vegetable matrices (including chlorophyll, vegetable proteins, and soluble sugars) remain elusive.

To investigate the mechanism of matrix interference, this study deconstructed the parathion ELISA into three critical steps: antigen–antibody binding, antibody–IgG-HRP binding, and HRP-catalyzed reaction. The extent of interference caused by vegetable matrices (chlorophyll, protein, glucose, fructose, sucrose) was evaluated. Subsequently, the interference of vegetable matrix on parathion residue ELISA in vegetable samples was explored. The results of this work can provide a theoretical foundation for minimizing vegetable matrix interference on ELISA and establish a methodological basis for developing highly specific parathion ELISA.

## 2. Materials and Methods

### 2.1. Reagents and Instruments

Anti-parathion monoclonal antibody and parathion–bovine serum albumin (BSA) complete antigens were from Chinese Academy of Agricultural Sciences (Beijing, China). Goat anti-mouse immunoglobulin G/horseradish peroxidase (IgG-HRP) were from Jackson Immunology Research Laboratory. Parathion standard was from Dr. Ehrenstorfer GmbH (Augsburg, Germany). Acetonitrile and methanol (HPLC grade) were from Merck, Germany. 3,3′,5,5′-tetramethylbenzidine (TMB) single-component substrate solution were from Beijing Solarbio Science & Technology Co., Ltd. (Beijing, China). Chlorophyll AB mixture and perilla protein were obtained from Macklin Biochemical Co., Ltd. (Shanghai, China); glucose from Xilong Scientific Co., Ltd. (Shantou, China); and sucrose and fructose were from Sigma-Aldrich (St. Louis, MO, USA). A 96-well microtiter plate, BCA Protein Assay Kit, and ion chromatography system were from Thermo Fisher Scientific Inc. (Waltham, MA, USA). Microplate reader was obtained from Tecan Group Ltd. (Männedorf, Switzerland) and DEM-3 microplate washer from Top Instrument Co., Ltd. (Beijing, China). Microplate thermostatic shaker was from Lanjeke Technology Co., Ltd. (Hangzhou, China).

### 2.2. Analysis of Matrix Interference Mechanism

To investigate the interference mechanism of matrix on ELISA, this study deconstructed the parathion ELISA into three key steps: antigen–antibody binding, antibody–IgG-HRP binding, and HRP-catalyzed reaction. Chlorophyll, perilla protein, glucose, fructose, and sucrose were serially diluted with PBS (10% methanol) to generate a concentration gradient series and subsequently applied to study the interference mechanism on ELISA.

#### 2.2.1. Analysis of Matrix Interference on Antigen–Antibody Binding

To investigate the vegetable matrix interference on antigen–antibody binding, parathion–BSA complete antigen was added to a 96-well plate and incubated at 37 °C for 2 h. After washing, the plate was blocked with 2% BSA for 1 h. Following another washing step, serially diluted solutions of chlorophyll, perilla protein, glucose, fructose, and sucrose were mixed with the working concentration of anti-parathion antibody, respectively, and transferred to the 96-well plate for 2 h incubation. Post-washing, 100 μL of IgG-HRP was added to each well and incubated for 1 h. After a final washing step, 100 μL of TMB single-component substrate solution was added to each well for 15 min of color development at room temperature. The absorbance value was measured using a multifunctional microplate reader, and the I_m_ value was calculated.

#### 2.2.2. Analysis of Matrix Interference on Antibody–IgG-HRP Binding

To investigate the vegetable matrix interference on the antibody–IgG-HRP binding step, the working concentration of the anti-parathion antibody was first immobilized on a 96-well plate by incubation at 37 °C for 2 h. Then, the plate was blocked with 2% BSA for 1 h. After washing, solutions of chlorophyll, perilla protein, glucose, fructose, and sucrose were mixed with IgG-HRP and transferred to the 96-well plate for a 2 h incubation. Following another washing step, 100 μL of TMB single-component substrate solution was added and for a 15 min at room temperature. The absorbance value was measured using a multifunctional microplate reader, and the I_m_ value was calculated.

#### 2.2.3. Analysis of Matrix Interference on HRP

To investigate the direct interference of vegetable matrices on the catalytic activity of HRP, IgG-HRP was immobilized on a 96-well plate by incubation at 37 °C for 2 h. After washing, serially diluted solutions of chlorophyll, perilla protein, glucose, fructose, and sucrose were added to the wells and incubated for a 2 h incubation. Then, 100 μL of TMB single-component substrate solution was transferred to each well for 15 min of color development at room temperature. The absorbance was measured using a multifunctional microplate reader, and the I_m_ value was calculated.

In this study, the I_m_ value was utilized as a quantitative indicator to evaluate the level of matrix interference and calculated according to the following formula [29]:

I_m_ (%) = |OD_solvent_ − OD_test_|/OD_solvent_ × 100

where OD_solvent_ is the absorbance value of PBS (10% methanol) at 650 nm, and OD_test_ is the absorbance value of solutions (chlorophyll, perilla protein, glucose, fructose, sucrose) or sample extracts at 650 nm.

### 2.3. Elimination of Vegetable Matrix

To evaluate the elimination efficiency of matrix of acetic acid treatment, the chlorophyll and perilla protein was used as a representative vegetable matrix.

#### 2.3.1. Chlorophyll

Chlorophyll solution (18.21 mg·mL^−1^) was prepared in PBS (10% methanol). After adding 100 μL of acetic acid and leaving it for 5 min, the solution was centrifuged at 8000 rpm (4 °C) for 2 min and filtered through a 0.22 μm nitrocellulose membrane into a centrifuge tube. The treated chlorophyll solution was added to a 96-well plate (100 μL per well), and absorbance values were measured using a multifunctional microplate reader. Chlorophyll content (mg·mL^−1^) was calculated using the following formulas [30]:

Chlorophyll A = 12.72A_663_ − 2.59A_645_

Chlorophyll B = 22.88A_645_ − 4.67A_663_

Total Chlorophyll = Chlorophyll A + Chlorophyll B

where A_663_ is the absorbance value of test solution at 663 nm, and A_645_ is the absorbance value of test solution at 645 nm.

#### 2.3.2. Protein

To evaluate the cleanup efficiency of acetic acid treatment on protein, a perilla protein solution was prepared in PBS (10% methanol) as a representative vegetable protein matrix. After adding 100 μL of acetic acid and incubating for 5 min, the solution was centrifuged at 8000 rpm (4 °C) for 2 min and filtered again through a 0.22 μm nitrocellulose membrane into a centrifuge tube. Protein concentrations before and after acetic acid treatment were determined using the BCA assay.

### 2.4. Verification in Vegetable Samples

To investigate the vegetable matrix interference on residual parathion ELISA, the spike-and-recovery test with vegetable samples was conducted, and details of pre-treatment can be found in Supplementary Materials. The vegetable samples (spinach, perilla, and purple kale) were chosen as representative to highlight key matrix interference scenarios in this study, namely high chlorophyll content, high protein content, and high soluble sugar content, respectively. The practical vegetable samples were then subjected to different pre-treatments for subsequent ELISA.

### 2.5. Establishment of an Immunoassay

First, parathion–BSA complete antigen was added to the 96-well plate, which was incubated at 37 °C in a microplate thermostatic shaker for 2 h. After three washing cycles, 2% BSA (300 μL per well) was added to the wells and incubated at 37 °C for 1 h for blocking. Subsequently, 50 μL parathion solution was mixed with 50 μL of parathion monoclonal antibody and added to the 96-well plate for competitive reaction at 37 °C for 2 h. After three additional washes, 100 μL of IgG-HRP was added and incubated at 37 °C for 1 h to bind with the parathion monoclonal antibody. Finally, 100 μL of TMB single-component substrate solution was added to each well after final washing, followed by 15 min of color development at room temperature. The absorbance value was measured using a multifunctional microplate reader. Two parathion solvent standard curves were established with parathion concentration as the abscissa and inhibition rate as the ordinate.

### 2.6. Spike-and-Recovery Test for Vegetable Samples

The interference of vegetable matrices on the parathion ELISA was evaluated based on I_m_ values and average recovery rate. After weighing, vegetable samples were spiked with parathion at concentrations of 10, 50, and 100 μg·L^−1^, homogenized, and incubated for 1 h before pre-treatment. Extracts from blank pretreated samples were used as diluents to prepare parathion solutions for ELISA. A matrix-matched standard curve was established to calculate average recovery rates. To assess matrix interference in ELISA, the I_m_ value was determined by comparing the absorbance of blank sample extracts with that of PBS (10% methanol).

### 2.7. Statistical Analysis

The results of this study are presented as the mean ± standard deviation (SD) of triplicate measurements (*n* = 3). The relative standard deviation (RSD) was also calculated as a measure of precision. Statistical analysis and data visualization were performed using Origin (version 2021).

## 3. Results and Discussion

### 3.1. Optimal Concentrations of Parathion–BSA Complete Antigens and Antibody

**Figure 1:**
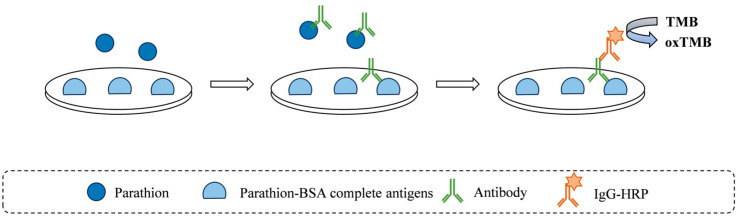
Scheme of ELISA for determining parathion.

A scheme of the parathion ELISA is presented in Figure 1. Determining the appropriate concentrations of coating antigen and antibody is a critical step in ELISA, directly influencing the sensitivity, specificity, reproducibility, and cost-effectiveness of the detection. As shown in Table 1, the absorbance value exhibited a significant upward trend with increasing antibody concentrations, while antigen concentration variations had minimal impact on absorbance. When antigen concentrations ranged between 0.50 and 3.00 mg·L^−1^ and antibody concentrations ranged between 1.00 and 2.00 mg·L^−1^, the absorbance values consistently fell within the range of 1 to 2. Based on the cost-effectiveness principle [31,32], this study selected an antigen concentration of 0.5 mg·L^−1^ and an antibody concentration of 1.00 mg·L^−1^ as the working concentrations for subsequent ELISA.

### 3.2. Optimization of Competitive Step Time

**Figure 2:**
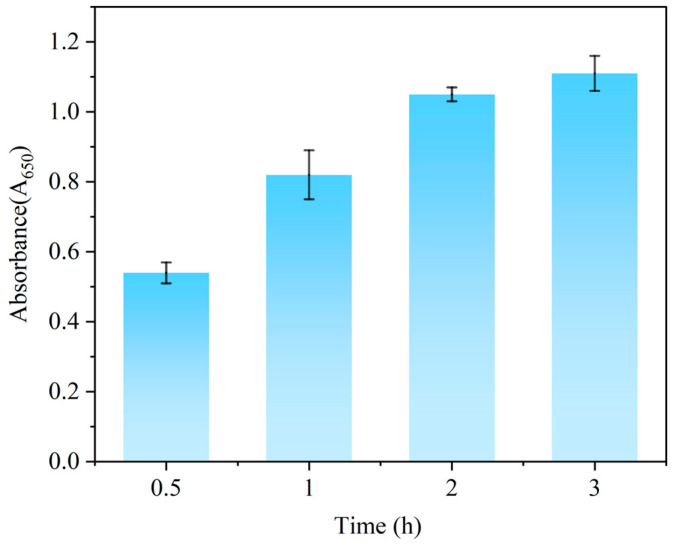
The absorbance value at different times of competitive step.

An appropriate competitive step time facilitates the efficiency of the immunoassay. In this study, the competitive step time was optimized by controlling the reaction duration. As illustrated in Figure 2, the absorbance value variation became negligible when the reaction time exceeded 2 h. Therefore, 2 h was determined as the optimal competitive reaction duration.

### 3.3. Vegetable Matrix Interference on Antigen–Antibody Binding

**Figure 3:**
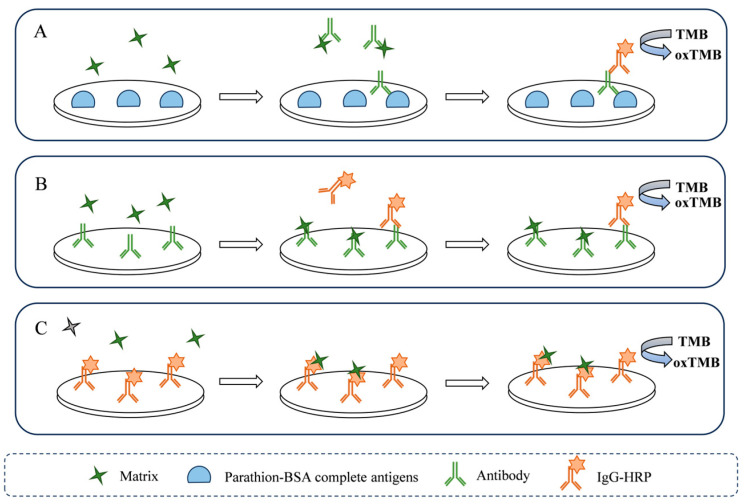
Illustration of matrix interference on three critical steps of ELISA. (**A**) Interference on antigen–antibody binding. (**B**) Interference on antibody–IgG-HRP binding. (**C**) Interference on HRP-catalyzed reaction.

**Figure 4:**
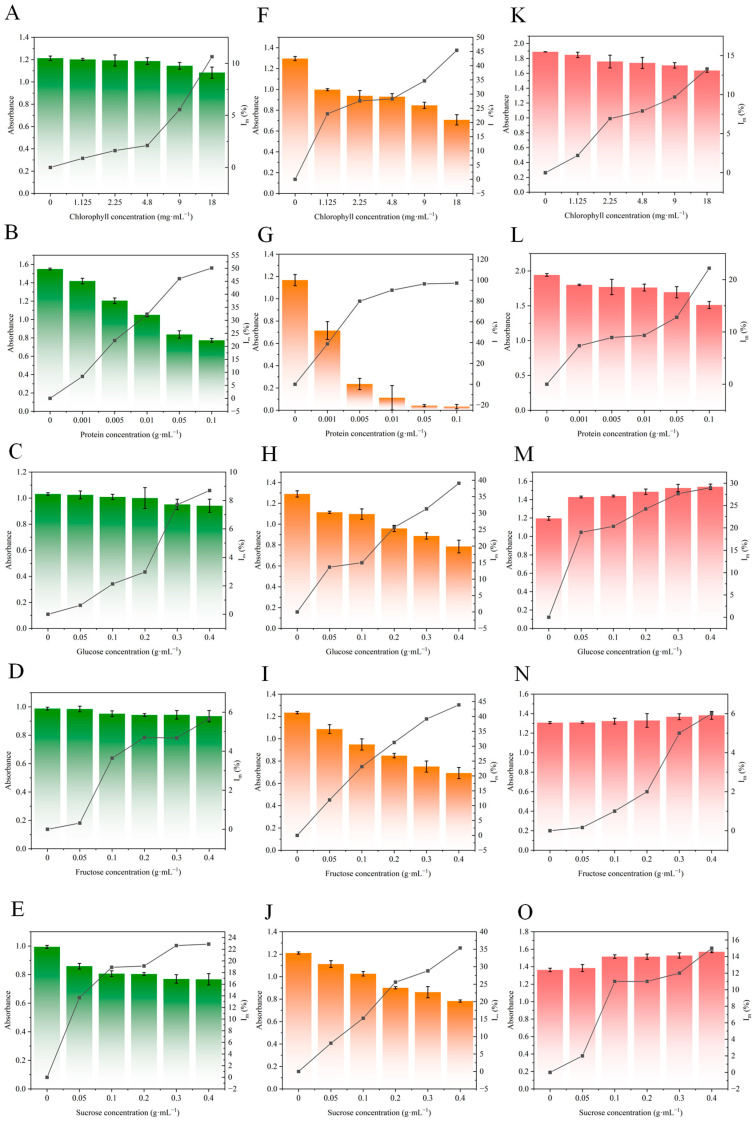
The interference of chlorophyll, perilla protein, sucrose, fructose, and glucose on ELISA. (**A**–**E**) vegetable matrix interference on antigen–antibody binding. (**F**–**J**) Vegetable matrix interference on antibody–IgG-HRP binding. (**K**–**O**) Vegetable matrix interference on HRP.

To investigate the vegetable matrix interference on antigen–antibody binding, serially diluted solutions of chlorophyll, perilla protein, glucose, fructose, and sucrose were added to the antigen–antibody binding process (Figure 3A). Following the addition of matrix to the reaction, the absorbance decreased with increasing matrix proportion, exhibiting a positive correlation between the I_m_ and matrix proportion (Figure 4A–E). Notably, the reaction incorporating perilla protein exhibited a pronounced reduction in absorbance. Even at the lowest tested concentration of perilla protein (0.001 g·mL^−1^), the I_m_ reached 10%. This significant interference is likely attributable to nonspecific binding that occupies antigen–antibody recognition sites and introduces steric hindrance. Proteins are frequent sources of matrix interference. Previous studies on protein interference have consistently indicated the potential for interactions between proteins and antibodies to cause anomalous assay results [33,34]. Furthermore, vegetable proteins, such as storage proteins, globulins, and glutenin [35,36], can bind non-specifically to the fragment antigen binding of antibody. This binding not only results in occupation of antigen-binding sites but also causes significant steric hindrance [37].

High concentrations of soluble sugars elevate sample viscosity, leading to a crowded microenvironment that impedes molecular diffusion during antigen–antibody and antibody–IgG-HRP binding [38]. As shown in Figure 4E, sucrose exerted a greater degree of interference on the antigen–antibody binding than glucose and fructose. At the same minimum concentration (0.05 g·mL^−1^), sucrose yielded an I_m_ of 13%, exceeding the I_m_ of glucose (0.6%) and fructose (0.3%). This difference may stem from sucrose’s disaccharide structure, contrasting with the monosaccharide nature of glucose and fructose. The greater molecular mass of the disaccharide consequently induces more pronounced steric hindrance via nonspecific adsorption during the antigen–antibody binding process.

### 3.4. Vegetable Matrix Interference on Antibody–IgG-HRP Binding

To investigate the vegetable matrix interference on antibody–IgG-HRP binding, serially diluted solutions of chlorophyll, perilla protein, glucose, fructose, and sucrose were added to the antibody–IgG-HRP binding process (Figure 3B). Similar to the antigen–antibody binding step, the absorbance decreased with increasing matrix proportion, exhibiting a positive correlation between the I_m_ and matrix proportion (Figure 4F–J). It is noteworthy that, in the presence of the same matrix, the I_m_ of the antibody–IgG-HRP binding step was significantly higher than that of the antigen–antibody binding step. At a chlorophyll concentration of 1.125 mg·mL^−1^, the I_m_ for antibody–IgG-HRP binding reached 23%, markedly higher compared to antigen–antibody binding (1%). Similarly, at a protein concentration of 0.001 g·mL^−1^, the I_m_ for antibody–IgG-HRP binding was 39%, far exceeding values for antigen–antibody binding (8%).

According to the analysis based on the structure of the matrix, chlorophyll consists of a porphyrin ring head and a phytol tail [39]. The porphyrin moiety can nonspecifically bind to amino acids in proteins [40], while Mg^2+^ in chlorophyll forms coordination bonds with histidine (His) residues in proteins, enhancing nonspecific interactions [41,42]. Notably, antibodies contain a high density of His residues, predominantly located in the fragment crystallizable region that binds with IgG-HRP [43,44]. This is why antibody–IgG-HRP binding is more susceptible to chlorophyll interference compared to antigen–antibody binding.

The observation that proteins exert greater interference on antibody–IgG-HRP binding than on antigen–antibody binding may be attributed to enhanced nonspecific binding between IgG-HRP and proteins. Unlike antibodies, IgG-HRP is generated through chemical conjugation of HRP to functional groups on the IgG surface. Modifications to these surface functional groups can substantially alter protein adsorption properties [45]. Consequently, it is plausible that the conjugation process induces conformational changes or alters charge distribution, with this structural modification significantly promoting nonspecific interactions between IgG and proteins. Furthermore, the concomitant presence of matrix and the IgG-HRP not only impedes antibody–IgG-HRP binding but also interferes with the catalytic activity of HRP. This dual interference mechanism explains why antibody–IgG-HRP binding exhibits markedly more pronounced matrix interference compared to antigen–antibody binding.

### 3.5. Vegetable Matrix Interference on HRP

To investigate the vegetable matrix interference on the enzymatic activity of HRP, serially diluted solutions of chlorophyll, perilla protein, glucose, fructose, and sucrose were mixed with the HRP (Figure 3C). Similar to the antigen–antibody binding step and antibody–IgG-HRP binding step, I_m_ decreased with increasing matrix proportion (Figure 4K–O). Intriguingly, absorbance in the HRP catalytic exhibited a progressive increase with rising concentrations of soluble sugars. This phenomenon may arise from nonenzymatic redox reactions involving reducing sugars [46], which directly participate in the chromogenic process by nonspecifically binding to antibody or adsorbing onto the microplate, thereby interfering with the accuracy of ELISA.

### 3.6. Vegetable Matrix Interference on Residual Parathion ELISA

To investigate the vegetable matrix interference on residual parathion ELISA, we conducted fortified recovery experiments using vegetable samples and compared the I_m_ and recovery rate before and after further elimination of the matrix.

Organic solvents can interfere with antibody–antigen binding specificity and enzymatic catalytic activity. For instance, organic solvents can disrupt hydrophobic interactions in protein tertiary structures, leading to conformational changes in antibody complementarity-determining regions (CDRs) or destabilizing the microenvironment of enzyme active sites. Antibody recognition capacity and enzyme catalytic efficiency are significantly reduced [47]. In a preliminary study, we confirmed that acetonitrile significantly compromises the accuracy and sensitivity of the parathion ELISA (Figure S1). In contrast, methanol demonstrates lower interference and is better suited for parathion ELISA. Thus, methanol was employed as the extraction solvent for vegetable samples.

Vegetable samples typically contain complex matrices that significantly interfere with ELISA. Notably, characteristic vegetable matrix such as chlorophyll and proteins exhibit instability under acidic conditions [48,49]. The chlorophyll molecule features a porphyrin ring core. Under acidic conditions, H^+^ replaces the central Mg^2+^ in the porphyrin ring, leading to the formation of pheophytin. This conformational change exposes hydrophobic groups, enhances overall molecular hydrophobicity, and ultimately induces molecular aggregation and precipitation [50]. To validate the cleanup efficacy of the acetic acid treatment for chlorophyll, chlorophyll solution was used to simulate green vegetable sample extracts, and concentration changes were compared before and after acetic acid treatment. After acetic acid treatment, the chlorophyll solution gradually turned yellow, with visible aggregated precipitates formed after centrifugation. Filtration through a 0.22 μm nitrocellulose membrane removed the precipitates, yielding a clarified solution. Absorbance value at 645 nm and 663 nm revealed a reduction in chlorophyll concentration from 18.61 mg·L^−1^ to 1.41 mg·L^−1^ (Figure S2A), demonstrating the effectiveness of acetic acid treatment in reducing chlorophyll concentration.

To evaluate the cleanup effect of acetic acid treatment on protein, perilla protein was employed as a representative vegetable protein. Under alkaline conditions, peptide bonds in perilla protein reduce Cu^2+^ to Cu^+^, which binds to BCA reagents to form a stable purple complex with maximum absorbance at 562 nm [51]. Using this principle, the BCA assay was applied to measure absorbance value at 562 nm for perilla protein before and after acetic acid treatment, centrifugation, filtration, and pH adjustment to neutrality. As shown in Figure S2B, after acetic acid treatment, the absorbance value decreased compared to non-acid treatment. Since absorbance value intensity correlates with protein concentration, these results confirm that acetic acid treatment reduces protein concentration.

This study leveraged the instability of vegetable matrix under acidic conditions. During pretreatment of spinach, perilla, and purple cabbage, acetic acid treatment was incorporated to further purify matrices, thereby investigating vegetable matrix interference on residual parathion ELISA. At the same time, the acetic acid treatment was compared with the non-acid treatment and instrumental detection methods to investigate vegetable matrix interference on residual parathion ELISA. Vegetable matrix interference on parathion ELISA was evaluated based on I_m_ and average recovery rate. As shown in Table 2 and Figure S3, compared to the I_m_ of non-acid treatment vegetable samples (16–26%), the acetic acid treatment vegetable sample yielded more satisfactory I_m_ (10–13%), indicating that acetic acid treatment reduced matrix interference levels. Additionally, as matrix interference decreased, the recovery rates of parathion in vegetable samples became more comparable to the results from instrumental methods. The average recovery rate of spinach samples was adjusted from 87 to 144% to 84–123%, perilla from 127 to 178% to 108–128%, and purple kale from 217 to 225% to 80–120%. The changes in I_m_ values and recovery rates corroborate that vegetable matrix have significant interference on ELISA for parathion.

## 4. Conclusions

Vegetables matrix severely interferes with ELISA, significantly restricting its application in pesticide residue detection. This study investigated the interference mechanism of vegetable matrices on ELISA and quantitatively assessed their interference intensity using the I_m_. The results demonstrate that vegetable matrix (chlorophyll, proteins, glucose, fructose, sucrose) substantially interferes with the ELISA step of antigen–antibody binding, antibody–IgG-HRP binding, and HRP catalysis. Notably, the antibody–IgG-HRP binding step was subject to the most pronounced interference. Conversely, soluble sugars enhanced the chromogenic reaction, thereby generating false-positive signals. Collectively, matrix interference undermines the reliability of ELISA for pesticide residue detection, as evidenced by the I_m_ and recovery rates of parathion residue ELISA in differentially pre-treated vegetable samples. Non-acetic acid-treated vegetable samples exhibited high I_m_. Following acetic acid treatment, with the reduction of I_m_, the recovery rate of parathion in vegetable samples was also more satisfactory. These findings provide a theoretical foundation for minimizing vegetable matrix interference on ELISA and establish a methodological basis for developing highly specific parathion ELISA. It should be noted that the effectiveness of this acetic acid treatment is contingent upon the instability of the matrix components under acidic conditions; for matrices that are acid-stable, the cleanup effect may be limited. Future research should focus on understanding the interactions between the vegetable matrix and recognition/signal elements, alongside conformational changes of these elements within the matrix, to fully comprehend the vegetable matrix interference on ELISA.

## Figures and Tables

**Table 1:** Selection of antigen and antibody concentrations.

Antibody (mg·L^−1^)	Parathion-BSA Complete Antigens (mg·L^−1^)			
	0.50	1.00	2.00	3.00
0.25	0.44	0.67	0.53	0.56
0.50	0.62	0.66	0.69	0.65
1.00	1.00	1.09	1.06	1.05
2.00	1.62	1.76	1.76	1.68
4.00	2.40	2.56	2.60	2.57
8.00	2.81	3.04	3.14	2.97

**Table 2:** Parathion recovery rate and I_m_ value of vegetable samples.

Method	Sample	Pre-Treatment	Average Recovery Rate/%	I_m_/%	RSD/%	Ref.
ELISA	Spinach	Non-acid treatment	87–144	16	1–10	This study
	Perilla		127–178	10	0–13	
	Purple kale		217–225	26	2–6	
	Spinach	Acetic acid treatment	84–113	14	1–7	
	Perilla		80–111	23	1–8	
	Purple kale		82–120	13	2–6	
Gas chromatography	Spinach	Magnetic solid-phase extraction	97–98	/	/	[52]
	Lettuce		101–121	/	/	
	Cabbage		102–114	/	/	
Negative corona discharge-ion mobility spectrometer	Celery	Solid-phase microextraction	85–90	/	5–11	[53]
High-performance liquid chromatography	Tomato	Solid-phase microextraction	92–96	/	5–7	[54]
	Cabbage		86–92	/	6–6	
	Barley		86–96	/	4–7	

Note: The symbol “/” indicates that the value was not measured or reported in the corresponding reference.

## Data Availability

The original contributions presented in the study are included in the article/Supplementary Materials, further inquiries can be directed to the corresponding author.

## Supplementary Materials

The following supporting information can be downloaded at https://www.mdpi.com/article/10.3390/foods14193414/s1, Figure S1: Parathion ELISA solvent standard curve. (A) Acetonitrile; (B) Methanol; Figure S2: Changes in (A) chlorophyll concentration and (B) protein absorbance values before and after acetic acid-treatment; Figure S3: Matrix-matched calibration curve of parathion in (A) spinach (B) perilla (C) purple kale.
