# Supplementary material for: Matrix Interference of Vegetable on Enzyme-Linked Immunosorbent Assay for Parathion Residue Detection
Source: Foods. 2025 Oct 3;14(19):3414. doi: 10.3390/foods14193414 (PMC12524109; doi:10.3390/foods14193414)
Supplement: Supplementary file 1 [file foods-14-03414-s001.zip › foods-3876409-supplementary.pdf]

Supporting Information

Matrix interference of vegetable on enzyme-linked immunosorbent assay for parathion residue detection

Linglong Chena,b,1, Ge Chena,1 *, Xing Zhanga, Qinghuan Wua,b, Guangyang Liua, Xiaomin Xua, Yanguo Zhanga, Lingyun Lia, Lin Qina, Jing Wangc, Maojun Jinc, Donghui Xua and *

a Institute of Vegetables and Flowers, Chinese Academy of Agricultural Sciences, State Key Laboratory of Vegetable Biobreeding, Key Laboratory of Vegetable Quality and Safety Control, Ministry of Agriculture and Rural Affairs of China, Ministry of Agriculture Vegetable Product Quality Safety Risk Assessment Laboratory, Beijing, 100081, P. R. China
b Chongqing Key Laboratory of Olericulture, College of Horticulture and Landscape Architecture, Southwest University, Chongqing, 400715, China
c Institute of Quality Standard and Testing Technology for Agro-Products, Chinese Academy of Agricultural Sciences, Key Laboratory of Agro-Product Quality and Safety, Ministry of Agriculture and Rural Affairs of China 100081, P. R. China
Correspondence
Ge Chen, Chinese Academy of Agricultural Sciences, Institute of Vegetables & Flowers, No. 12, Southern Street of Zhongguancun, Haidian District, Beijing 100081, China
E-mail: xudonghui@caas.cn; chenge@caas.cn

1. Optimization of organic solvents

To identify a suitable organic solvent, parathion stock solutions were prepared using acetonitrile and methanol, and solvent-based standard curves were established with the logarithm of parathion concentration as the X-axis and inhibition rate as the Y-axis (Figure S1).

The acetonitrile-based standard curve exhibited a linear equation of $y = 0.4266x - 0.3149$ ($R^2 = 0.9315$), indicating poor linearity, with a limit of detection (LOD, corresponding to 10% inhibition rate) of 9.39 μg·L-1 and a IC50 (sensitivity, 50% inhibition rate) of 81.32 μg·L-1. In contrast, the methanol-based standard curve demonstrated superior linearity, with a linear equation of $y = 0.4681x - 0.2682$ ($R^2 = 0.9894$), a decrease in LOD to 6.11 μg·L-1, and a decrease in IC50 to 43.76 μg·L-1. Based on these findings, methanol was selected as the solvent for subsequent ELISA and sample extraction to minimize organic solvent-induced interference.

2. Non-acetic acid-treatment

Samples were washed and homogenized thoroughly. 10 g of homogenized samples were freeze-dried in -70°C lyophilizer. Subsequently, the freeze-dried sample was mixed with 10 mL methanol, vortexed for 1 min, and centrifuged at 6000 rpm (4°C) for 5 min. 2 mL of the supernatant was transferred to a cleanup tube containing 130 mg of dSPE sorbents (52 mg ethylenediamine-N-propylsilane, 52 mg C18, and 26 mg graphitized carbon black), vortexed for 1 min, and centrifuged again at 6000 rpm (4°C) for 2 min. Finally, the supernatant was filtered through a 0.22 μm nitrocellulose membrane into a centrifuge tube, 9 mL of PBS was added and stored at 4°C.

3. Acetic acid-treatment

Samples were washed and homogenized thoroughly. 10 g of homogenized samples were freeze-dried in -70°C lyophilizer. Subsequently, the freeze-dried sample was mixed with 10 mL methanol, vortexed for 1 min, and centrifuged at 6000 rpm (4°C) for 5 min. 2 mL of the supernatant was transferred to a cleanup tube containing 130 mg of dSPE sorbents (52 mg ethylenediamine-N-propylsilane, 52 mg C18, and 26 mg graphitized carbon black), vortexed for 1 min, and centrifuged again at 6000 rpm (4°C) for 2 min. Finally, 1 mL of the nitrocellulose-filtered extract was combined with 9 mL PBS and 100 μL acetic acid, incubated for 5 min, and centrifuged at 8000 rpm (4°C) for 2 min. The supernatant was filtered again through a 0.22 μm nitrocellulose membrane into a centrifuge tube, adjusted to pH 6-7 with an appropriate amount of K2CO3, and stored at 4°C.

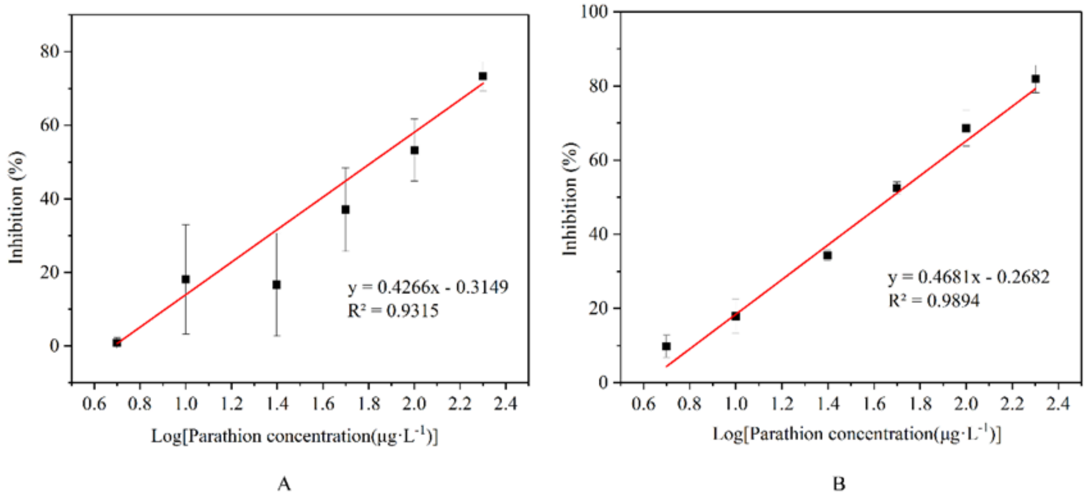

Figure S1. Parathion ELISA solvent standard curve. (A) Acetonitrile; (B) Methanol.

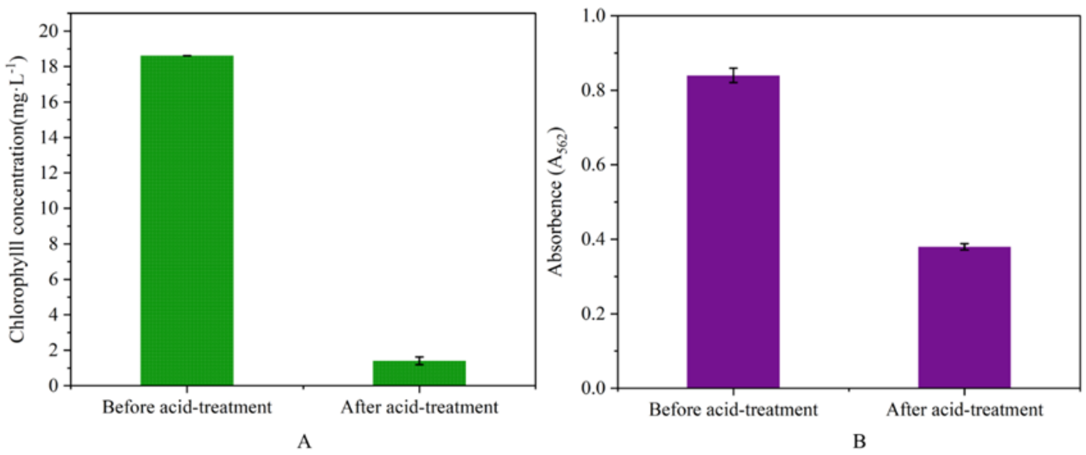

Figure S2. Changes in (A)chlorophyll concentration and (B)protein absorbance values before and after acetic acid-treatment.

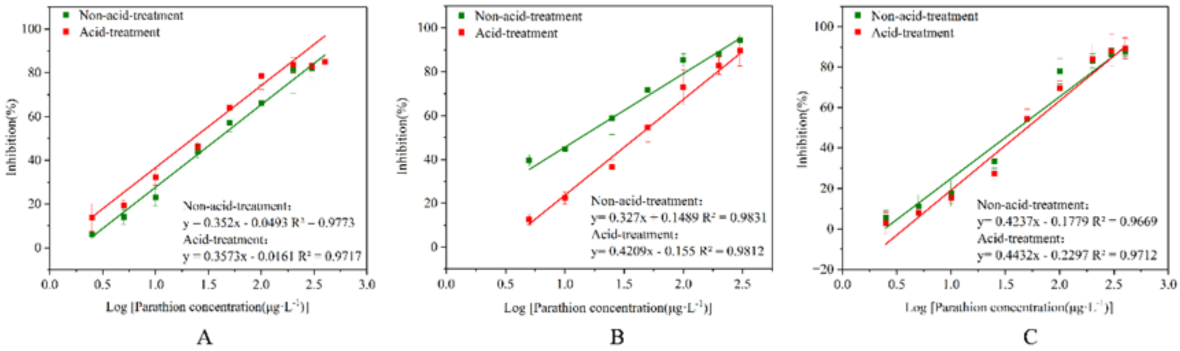

Figure S3. Matrix-matched calibration curve of parathion in (A) spinach (B) perilla (C) purple kale.
